# To Know Behavior Is to Know Ecology and Physiology: Integration and the Flexible Phenotype

**DOI:** 10.1371/journal.pbio.1001055

**Published:** 2011-05-03

**Authors:** Keith W. Sockman

**Affiliations:** Department of Biology and Curriculum in Neurobiology, University of North Carolina at Chapel Hill, Chapel Hill, North Carolina, United States of America

**Figure:**
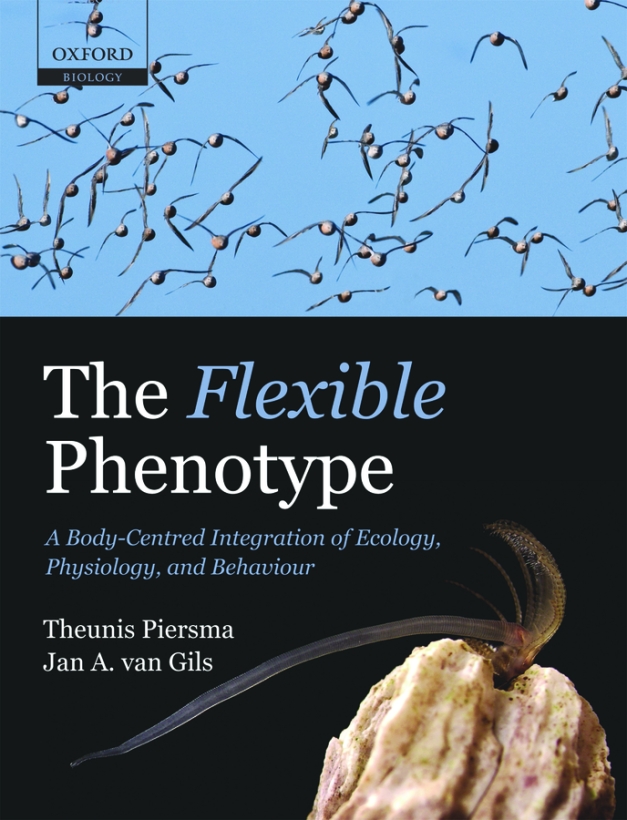
Piersma T, van Gils JA (2010) The Flexible Phenotype: A Body-Centred Integration of Ecology, Physiology, and Behaviour. New York: Oxford University Press. 248 p. ISBN 978-0199233724 (hardcover). US$117.00.

**About the Author**

Dr. Keith W. Sockman is an Associate Professor in the Department of Biology and Curriculum in Neurobiology at the University of North Carolina at Chapel Hill. He completed his PhD in Zoology in 2000 at Washington State University and conducted post-doctoral work at The Johns Hopkins University. Throughout his career, he has maintained an interest in the interface between ecology, behavior, and physiology and has used birds as a study system for his research. Recently, he has focused these interests on neurobiology and how, mechanistically, the brain integrates ecological and social information during reproductive decision-making.

Within most multicellular species, it is variation within individuals in how the genome is expressed, not variation between individuals in the genome itself that produces the larger, more rapidly induced phenotypic variability. For example, in many temperate-zone bird species, the testes increase in size by ∼100-fold over the course of a few weeks with the annual onset of the breeding season, a rate and magnitude of change within individuals that vastly exceeds the variation between individuals of the same species examined at the same stage of breeding. Feeding in some snakes elicits a change in digestive physiology and morphology that can include a doubling in liver, pancreas, and intestine mass and changes in stomach pH from 7–8 to 1–2, all within a week or so. Massive phenotypic variation. Zero genomic variation.

These examples of phenotypic change are impressive in their magnitude as well as their speed, both of which influence the accessibility of selection. But these rates of change are relatively modest compared to the speed with which behavior can drive phenotypic change in animals. Behavior, as a field of study, has provided a perspective on phenotypic variation that few other disciplines, including epigenetics, can match, yet it has been through its integration with other levels of biological organization that has enabled the most insight. Just as architects consider both the surrounding physical environment as well as the eventual occupants in designing a building, behaviorists must consider both the ecological context and the developmental and physiological mechanisms that give rise to the behavior. As Niko Tinbergen suggested in 1963, behavior cannot be completely understood in the absence of understanding the forces that ultimately and proximately influence it, and it is therefore no wonder why behavioral research that integrates across multiple levels of biological organization has been so well received by the larger biological research community in recent times.

The integration of multiple levels of biological organization combined with the importance of intra-individual phenotypic flexibility together serve as general themes throughout *The Flexible Phenotype: A Body-Centred Integration of Ecology, Physiology, and Behaviour*, a new book by Theunis Piersma and Jan A. van Gils. Piersma and van Gils take the reader through a loose history of their research program on the migration stop-over ecology, behavior, and physiology of the red knot (*Calidris canutus*), a migratory shorebird that has served as the foundation for their long-time collaboration. Although they load their story with examples and anecdotes from numerous other species, it is primarily the focus on the red knot that has enabled these authors to understand not only migratory stop-over biology, but more generally behavior, ecology, and digestive physiology as a whole. The writing style is more conversational than that to which we are typically accustomed as readers of the scientific literature. But beneath such whimsical section titles as “Thermometers Do Not Measure Feelings” and “It Takes Guts to Eat Shellfish” is a foundation of scientific rigor.

Part I of the book, “Basics of Organismal Design,” details the principles of water, heat, nutrient, and energy balance and explores the concept of symmorphosis, which posits that organisms are economically designed. Among the several intriguing ideas discussed in this section was one on the evolution of endothermy raised by Marcel Laassen and Bart Nolet in 2008. The adaptive basis for the evolution of endothermy has confounded researchers for decades, but, over the years, many converged on the idea that the elevated activity levels enabled by endothermy allowed better exploitation of relatively less active herbivores as a food resource. In other words, endothermic carnivores could more easily prey on herbivores. Taking an integrative approach by combining digestive physiology with foraging ecology, Laassen and Nolet hypothesized that endothermy evolved as a mechanism for burning the excess carbon consumed as part of a primarily herbivorous diet. Thus, endothermy may have first evolved in herbivores, not carnivores. Although researchers are sure to debate the evolutionary origins of endothermy for years to come, the utility of an integrative approach in this example was clear.

Part II, “Adding Environment,” begins by discussing the relationships between metabolic rate, activity, and performance. But it is not until the authors integrate ecology into their thinking that questions regarding performance limits can be satisfactorily answered, because it is the ecological environment that governs the severity of the punishment for over-exertion, either in terms of survival or reduced reproductive value. The concept of phenotypic flexibility is detailed in the next chapter, which offers many examples and provides insight from the authors as well as others on the adaptive significance of phenotypic flexibility. Of particular interest is the discussion on the 2007 book *The Tinkerer's Accomplice*, in which author J. Scott Turner builds on previous reasoning from Stephen Jay Gould and Richard Lewontin that most of an organism's phenotype is due not to natural selection on particular genes, but rather to “agents of homeostasis,” the self organization that follows from how developing structures interact with their immediate environment—be it the internal physiological and anatomical environment or the external physical, ecological, and social environment. A remarkable example is provided in the annual growth and shedding of antlers in deer. Antlers don't just get larger with each year's new growth, they can also retain certain “memories” of previous years' damage. That is, an antler that incurs velvet damage during its growth one year will retain evidence of that damage in subsequent years, even though the original damaged antler had long since been shed. Although the phenomenon is surprising, a probable mechanism rooted in the “memories” of neurons that innervate the velvet is not. Indeed, the internal regulator of most behavioral, physiological, and even anatomical plasticity in vertebrates is the nervous system. Because the vertebrate brain is the primary integrator of environment, physiology, and behavior, behavioral flexibility and the physiological and morphological flexibility of the body periphery cannot be completely understood in the absence of understanding neuroplasticity. It is therefore a bit surprising that Piersma and van Gils only occasionally mention the fundamental role of the nervous system in regulating most forms of organismal plasticity.

In “Adding Behaviour,” Piersma and van Gils introduce the details of their model system, the red knot, particularly as it pertains to optimal foraging and digestive plasticity, all the while underscoring the importance of integration and also of using natural, field-based systems for answering questions about phenotypic flexibility. In doing so, they introduce the concept of Bayesian updating, in which an individual combines prior information with new information to modify its behavioral decision, presumably in an adaptive fashion. This process requires the formation, consolidation, and retrieval of memories, none of which are possible in vertebrates without neuroplasticity.

Finally, in “Towards a Fully Integrated View,” the authors provide perhaps the most compelling rationale for the importance of integration in understanding behavior. Much of what makes it so compelling are the examples, in particular one, in which researchers discovered how the psychology of elk can reorganize an ecosystem. The fear of wolves (not necessarily the presence of wolves) drives elk from some areas that may afford little protection, thus releasing the streamside growth of aspen and willow that otherwise would be stunted by grazing. The presence of these mature trees provides a resource for beavers, whose dams cause the streams to meander and generate a mosaic of biodiverse microhabitats for everything from birds to butterflies. The conclusions: elk paranoia elevates butterfly diversity…and integration is essential.

This book is not simply a list of examples of how integration has helped us understand some behavioral problem; rather, it is more of a guide for using integration to investigate behavior as a vehicle for phenotypic flexibility. The integration that facilitates this process is difficult to practice. For the work of Piersma and van Gils, it requires expertise in physiology, behavior, and ecology, and, as these authors point out, attempting to be a jack of all of these trades runs the risk of mastering none of them. With their new book, Piersma and van Gils clearly demonstrate mastery not only at the three components of their integration, but also at the very process of integration, which is long overdue to be recognized as a trade in and of itself.

